# The Geographic Information System applied to study schistosomiasis in Pernambuco

**DOI:** 10.11606/S1518-8787.2017051000069

**Published:** 2017-11-13

**Authors:** Verônica Santos Barbosa, Rodrigo Moraes Loyo, Ricardo José de Paula Souza e Guimarães, Constança Simões Barbosa

**Affiliations:** I Fundação Oswaldo Cruz. Instituto Aggeu Magalhães. Recife, PE, Brasil; II Ministério da Saúde. Secretaria de Vigilância à Saúde. Instituto Evandro Chagas. Ananindeua, PA, Brasil; III Instituto Aggeu Magalhães. Departamento de Saúde Coletiva. Programa de Pós-Graduação em Saúde Coletiva. Recife, PE, Brasil

**Keywords:** Schistosomiasis, epidemiology, *Biomphalaria*, parasitology, Risk Factors, Geographical Localization of Risk, Geographic Information Systems, utilization, Esquistossomose, epidemiologia, *Biomphalaria*, parasitologia, Fatores de Risco, Localização Geográfica de Risco, Sistemas de Informação Geográfica, utilização

## Abstract

**OBJECTIVE:**

Diagnose risk environments for schistosomiasis in coastal localities of Pernambuco using geoprocessing techniques.

**METHODS:**

A coproscopic and malacological survey were carried out in the Forte Orange and Serrambi areas. Environmental variables (temperature, salinity, pH, total dissolved solids and water fecal coliform dosage) were collected from *Biomphalaria* breeding sites or foci. The spatial analysis was performed using ArcGis 10.1 software, applying the kernel estimator, elevation map, and distance map.

**RESULTS:**

In Forte Orange, 4.3% of the population had *S. mansoni* and were found two *B. glabrata* and 26 *B. straminea* breeding sites. The breeding sites had temperatures of 25ºC to 41ºC, pH of 6.9 to 11.1, total dissolved solids between 148 and 661, and salinity of 1,000 d. In Serrambi, 4.4% of the population had *S. mansoni* and were found seven *B. straminea* and seven *B. glabrata* breeding sites. Breeding sites had temperatures of 24ºC to 36ºC, pH of 7.1 to 9.8, total dissolved solids between 116 and 855, and salinity of 1,000 d. The kernel estimator shows the clusters of positive patients and foci of *Biomphalaria*, and the digital elevation map indicates areas of rainwater concentration. The distance map shows the proximity of the snail foci with schools and health facilities.

**CONCLUSIONS:**

Geoprocessing techniques prove to be a competent tool for locating and scaling the risk areas for schistosomiasis, and can subsidize the health services control actions.

## RESUMO

**OBJETIVO:**

Diagnosticar ambientes de risco para esquistossomose em localidades litorâneas de Pernambuco utilizando técnicas de geoprocessamento.

**MÉTODOS:**

Foi realizado inquérito coproscópico e malacológico nas localidades Forte Orange e Serrambi. Foram coletadas variáveis ambientais (temperatura, salinidade, pH, sólidos totais dissolvidos e dosagem de coliformes fecais da água) relacionadas aos criadouros ou focos de *Biomphalaria*. A análise espacial foi realizada no *software* ArcGis 10.1, aplicando-se o estimador kernel, mapa de elevação e mapa de distância.

**RESULTADOS:**

No Forte Orange, 4,3% da população estava com *S. mansoni* e existem dois criadouros de *B. glabrata* e 26 de *B. straminea*. Os criadouros apresentaram temperaturas de 25ºC a 41ºC, pH de 6,9 a 11,1, sólidos totais dissolvidos de 148 a 661 e salinidade de 1.000 d. Em Serrambi, 4,4% da população estava com *S. mansoni* e há sete criadouros de *B. straminea* e sete de *B. glabrata*. Os criadouros apresentaram temperaturas de 24ºC a 36ºC, pH de 7,1 a 9,8, sólidos totais dissolvidos de 116 a 855 e salinidade de 1.000 d. O estimador de kernel mostra os aglomerados de pacientes positivos e de focos de *Biomphalaria*, e o mapa de elevação digital indica áreas de concentração de águas pluviais. O mapa de distância mostra a proximidade dos focos dos caramujos em relação às escolas e unidades de saúde.

**CONCLUSÕES:**

As técnicas de geoprocessamento se mostraram como importantes ferramentas para a localização e dimensionamento das áreas de risco para esquistossomose, podendo subsidiar as ações de controle por parte dos serviços de saúde.

## INTRODUCTION

Schistosomiasis is caused by the helminth *Schistosoma mansoni*, which has as intermediate host a freshwater snail class Gastropoda, family Planorbidae, genus *Biomphalaria*. Those snails are pulmonary animals with a flat shell and they may be directly influenced by the environment they live in and have developed several survival strategies^7^.

Among the abiotic factors that influence the snail’s survival rate we have: pH, salinity, temperature, and rainfall. They live in biotopes with pH between 6.0 and 9.0; in acid environments (pH lower than 5.6), they are unable to promote the calcium deposition for the shell development, which prevents their colonization. The level of tolerable salinity for these snails is equal to or lower than 0.50‰, although *B. glabrata* specimens have been found in water with salinity 15 times higher than the accepted maximum for freshwater habitats^24^. The temperature also influences the snails, since it stimulates the cercariae release, whose ideal temperature for their development is around 20°C to 26°C, being able to tolerate temperatures of 18°C to 41°C. Rainfall influences they density, determining the formation and expansion of breeding sites or foci^12^.

In Pernambuco, schistosomiasis mansoni is endemic in rural areas, but studies in the Pernambuco coast show human cases presenting the acute clinical form and snail foci with infection rates up to 31% in localities as Forte Orange (Itamaracá), Porto de Galinhas (Ipojuca), Carne de Vaca and Ponta de Pedras (Goiana), Piedade (Jaboatão), Janga and Pau Amarelo (Paulista), and Mangue Seco (Igarassu)^8^ ^,^ ^10^.

An important instrument to be used in the description and analysis of health situation and one that subsidizes the management actions are the Geographic Information Systems (GIS)^21^. Studies using GIS are useful for the precise location of environments with *S. mansoni* intermediate hosts and human cases of the disease^2^, in order to estimate the areas of greatest occurrence^16^ and to perform temporal analysis related to hospital admission and mortality due the disease^23^. These systems also allow the construction of risk maps to generate models for schistosomiasis transmission and to predict the scaling of it using the diagnosis of risk factors^15^.

Geoprocessing presents itself as a modern tool to disseminate the results of epidemiological investigations, allowing epidemiologists to understand the dynamics of diseases and their variations in space and time. In addition, the information generated can be easily understood and interpreted by professionals and health services users^14^. This study aimed to diagnose risk environments for schistosomiasis transmission in coastal localities of Pernambuco using geoprocessing techniques.

## METHODS

### Study Areas

**Figure 1:**
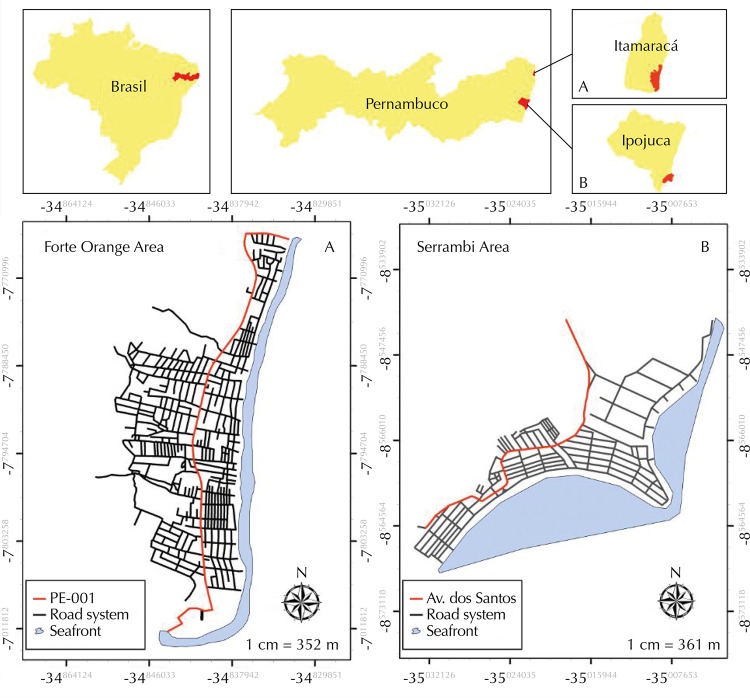
Spatial location of coastal localities: A – Forte Orange (Itamaracá) and B – Serrambi (Ipojuca), State of Pernambuco.

The study was carried out with the population served by the Local Helthcare Center (USF) of two coastal localities of Pernambucano (Figure 1): Forte Orange (A) and Serrambi (B).

Forte Orange is located south of Itamaracá Island, north coast of the state, and it is about 4 km long, limited to the east by the Atlantic Ocean and to the west by the highway PE-001 in a space 1.4 km wide. It is located 47.5 km from the city of Recife, being often visited by vacationers and tourists during the summer season.

Serrambi is located in the municipality of Ipojuca, being limited to the north by Cabo de Santo Agostinho, to the south by Sirinhaém, to the east by the Atlantic Ocean, and to the west by Escada. Located between Pontal de Maracaípe and Toquinho beach, Serrambi has 4 km long and 2 km wide coast. It is approximately 70 km away from the city of Recife.

### Coproscopic and Malacological Investigation

In 2013, a coproscopic survey was conducted on the population served by USF of Forte Orange and in 2014 in the USF population of Serrambi. The parasitological diagnosis was made in the Laboratory and Reference Service in Schistosomiasis (LASERE - IAM/FIOCRUZ), by the parasitological method Kato Katz (1972). The research was approved by the Instituto Aggeu Magalhães Research Ethics Committee (CAEE 07240712.6.0000.5190).

The malacological investigation was carried out by bimonthly collections during one year, considering the three climatic periods proposed by Barbosa et al.^6^ (dry season: from January to April, rainy season: from May to August, post-rain season: from September to December). Data were collected at the beginning and middle of each season. The collections were made with appropriate tools, like forceps, according to the depth of the water collection, following the method developed by Oliver and Schneiderman^20^. The species of the snails collected were identified by the technique of genital apparatus dissection^17^. The identification of the snail infection was carried out using the classical light exposure technique to stimulate the elimination of *S. mansoni* cercariae^25^ (a method to quantify infection intensity), and molecular diagnosis by Nested PCR – Polymerase Chain Reaction^1^, to detect the presence of *S. mansoni* DNA in the snails, a method that allows the identification of positivity, without informing the intensity of the infection.

### Environmental Variables Survey

In the localities of Forte Orange and Serrambi, environmental variables related to the *Biomphalaria* breeding sites or foci were collected during the malacological survey: temperature, salinity, pH, total dissolved solids (TDS), and fecal coliform dosage.

Procedures for data collection:

- Temperature, salinity, pH, and TDS of the water were measured during a malacological survey in loco with a digital spit thermometer, refractometer (0%–100% or parts per thousand – ppm and specific gravity or density 1,000–1,070 d), digital pH meter (0–14 scale), and digital TDS meter (0 to 999 ppm), respectively.
- Dosage of fecal coliforms: 100 ml of water from each breeding site or foci were collected during each malacological investigation to measure fecal contamination and then they were mixed with a Colitag™ chromogenic substrate for the detection of coliforms. The test was positive for fecal coliforms when the solution showed yellow and was positive for *Escherichia coli* when a bluish fluorescence was seen by subjecting the sample to a long wavelength ultraviolet light source. It was negative in the absence of color modification.

The characteristics of the breeding sites or foci were also observed in loco, such as: temporality, type, water level, the presence of vegetation, and contact with the population.

### Geoprocessing and Spatial Analysis

The localities sketches were constructed by vectoring the streets from images obtained in Google Earth using the TrackMaker software (http://www.trackmaker.com/). For the spatial location of the residence of the interviewed persons and the breeding sites or foci present in the localities, the absolute method was used with the instantaneous positioning a point, collected using a Global Positioning System (GPS) model Garmin Montana 650.

To identify areas with clusters of human positive cases and intermediate host snail breeding sites, we used the kernel density estimator, a nonparametric interpolation statistical technique that produces a continuous density surface calculated at all locations for the visual identification of hotspots^4^.

The kernel was estimated by the following equation:

$${\overset{⏜}{\lambda }}_{\tau }\left(S\right)=\sum_{i=1}^{n}\frac{1}{\tau^{2}}\kappa \left(\frac{\left(S-S_{i}\right)}{\tau }\right)$$

In the equation, ${\overset{⏜}{\lambda }}_{\tau }\left(S\right)$ is the estimated value in a region; *k*() is the kernel estimation function and *s*_1_ *,...,s*_n_ are locations of *n* observed events (samples) in a radius of influence τ with center in *s*.

The file generated by the kernel was a regular grid with spatial resolution (*x, y*) of 10 meters and with τ = 250 m, applied to the location data of patients with schistosomiasis and *S. mansoni* intermediate host snail foci.

We generate an elevation map to identify the area topography, showing rainwater accumulation sites which helps the establishment of intermediate host snail breeding sites. We used the digital elevation model (DEM), obtained from the Shuttle Radar Topography Mission (SRTM) with a spatial resolution of 90 meters.

To measure the proximity of the foci to the areas with the highest population influx, a distance map was created, obtained through the Euclidian Buffer, a technique that measures distances following a two-dimensional Cartesian plane. The Buffer was established considering the average distance that one individual can walk in one day to do his daily activities^3^. The coverage area was controlled by topography, which requires a reduction of displacement that shortens the distances^3^.

The spatial analysis was performed in the ArcGis 10.1 software (http://www.esri.com/), using the variables: residences with positive patients for schistosomiasis and foci of *S. mansoni* intermediate host snail.

## RESULTS

The stool examination was performed on 1,604 residents of Forte Orange USF and 69 (4.3%) were infected with *S. mansoni*. In 28 breeding sites, we collected 4,122 snails, of which 3,987 were *B. straminea* (in 26 breeding sites) and 135 were *B. glabrata* (in two breeding sites). The 26 breeding sites of *B. straminea*, 10 were positive for *S. mansoni* by the Nested PCR technique and two by the light exposure technique (rates of 2% and 0.7%). No *B. glabrata* breeding sites were positive for *S. mansoni*. Most of the breeding sites or foci were ditches, temporary, with low to medium water level, with low to high vegetation abundance, and intense contact with the population. The month of July presented the largest number of snails collected (1,451), only four collection sites showed contamination only with total coliforms and the remainder was positive for total coliforms and *E. coli*. The Table show the environmental data sampled from the breeding sites.

**Table:** Physico-chemical characteristics of breeding sites and foci.

Study area	Total breeding sites		Total foci		Temperature		pH		TDS		Salinity
				
	Bg	Bs	Bg	Bs	Min	Max	Min	Max	Min	Max	
Itamaracá	2	26	0	23	25^o^C	41^o^C	6.9	11.1	148	661	1,000 d
Serrambi	7	7	6	2	24^o^C	36^o^C	7.1	9.8	116	855	1,000 d

TDS: Total solids dissolved (in ppm); Bg: *B.glabrata*; Bs: *B.straminea*

**Figure 2:**
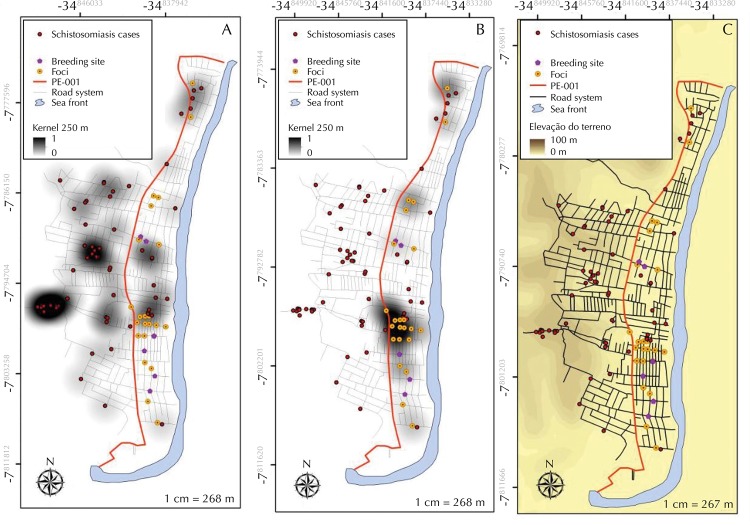
Kernel density estimator application in human cases (A), intermediate host snail foci (B), and digital elevation model (C). Forte Orange, Ilha de Itamaracá, State of Pernambuco.

Figure 2 shows the kernel density estimator results, showing the clusters of positive patients for schistosomiasis (A), the *Biomphalaria* foci (B), and the digital elevation model (C), showing the points of rainwater concentration that contribute to the establishment of intermediate host snail breeding sites or foci in Forte Orange.

At the Serrambi USF, 1,414 persons were examined and 63 (4.4%) were infected with *S. mansoni*. 14 breeding sites were identified and georeferenced, with 1,337 snails, of which 668 were *B. straminea* (in 7 breeding sites) and 669 were *B. glabrata* (in 7 breeding sites). Analyzing the *B. glabrata* breeding sites, two were positive for *S. mansoni* by the classical light exposure technique (rates of 4.4% and 50%) and three were positive by Nested PCR. The *B. straminea* breeding sites, two were positive by Nested PCR. Most of the breeding sites or foci were puddles, temporary, with low to medium water level, with low to high vegetation abundance, and moderate contact with the population. The month of August was the one with the highest number of snails collected (585), only one breeding site was positive only for total coliforms and the rest was positive for total coliforms and *E. coli*. The Table show the environmental data sampled from the breeding sites.

**Figure 3:**
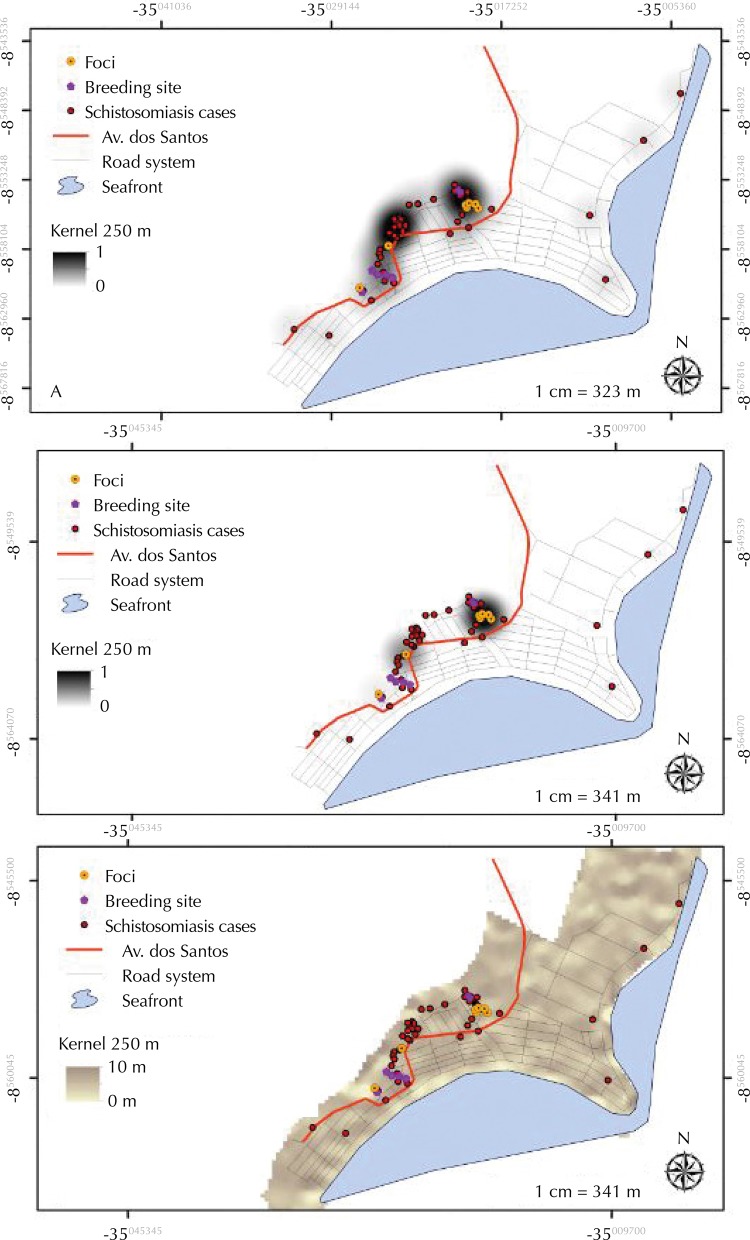
Kernel density estimator application in human cases (A), intermediate host snail foci (B), and digital elevation model (C). Serrambi, Ipojuca, State of Pernambuco.

Figure 3 shows the kernel density estimator results, showing the clusters of positive patients for schistosomiasis (A), the *Biomphalaria* foci (B), and the digital elevation model (C), showing the points of rainwater concentration that contribute to the establishment of intermediate host snail breeding sites or foci in Serrambi.

**Figure 4:**
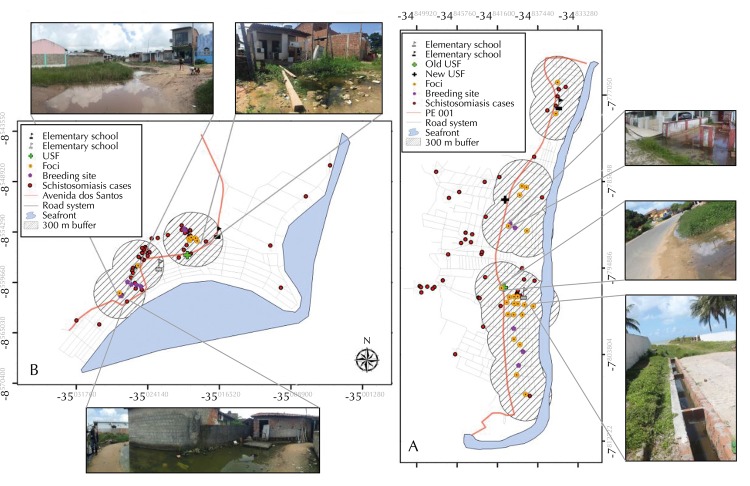
Proximal analysis of the schistosomiasis cases and intermediate host snails breeding sites and foci in relation to the main reference points of the locality A - Forte Orange (Itamaracá Island) and B - Serrambi (Ipojuca), State of Pernambuco.

The distance map (Figure 4) indicates the proximity of the intermediate host snail foci in relation to the schools and the Local Helthcare Center, places of great population influx.

USF: Local Health Center

## DISCUSSION

The Forte Orange beach was the first coastal area of Pernambuco to present human cases and intermediate host snails of *S. mansoni* ^18^. Since then, breeding sites and foci have been identified in this locality, initially *B. glabrata* ^5^ and later *B. straminea*. In Serrambi, *Biomphalaria* snails were only identified in 2015 but already showing an equivalent amount of *B. straminea* and *B. glabrata* ^13^.

The findings of this study reveal that *Biomphalaria* are increasingly adapted to adverse environmental conditions and remain viable for schistosomiasis transmission, a worrying factor that contributes to the establishment of the disease in new regions that until then would be hostile to the snail’s survival. Although they have been reported living at temperatures above 40°C^22^, there may be an interference in their growth rate, egg laying, survival, and intrinsic natural growth rate; furthermore, Barbosa et al.^12^ identified that, under laboratory conditions, exposure to a 42°C temperature was lethal for these snails. The *Biomphalaria* snails are also very sensitive to the pH variation of the environment, and until now, they have only been identified in environments with a pH up to 9,7^19^; this is the first report of *Biomphalaria* living at pH 11.1. TDS are composed of inorganic salts, organic matter, and other materials dissolved in water. The maximum limit of TDS for the living being survival is 500 mg/L. Changes in the ionic composition of water may exclude some species by promoting the growth of others; however, Silva^24^ identified breeding sites in Pernambuco with TDS up to 17,600 mg/L. The finding of total coliforms and *E. coli* in the breeding sites of the localities is a worrisome factor since the contamination of these environments is an important factor to maintain the schistosomiasis cycle in the localities.

The spatial distribution of schistosomiasis cases in Forte Orange is uniform, with two concentrations to the west of PE-001, the area most inhabited by natives and with the highest elevation. The foci were concentrated to the east of PE-001, where the abundant occurrence of the *B. straminea* species is observed, which is gradually occupying this area in the process of biological competition with *B. glabrata*, as shown by Barbosa et al.^9^ Most of the breeding sites and foci are temporary and artificial, formed by accumulation of water in the rainy season. Besides being conditioned by the climatic seasonality, they are in a lower slope area, a contributing factor to the water accumulation and permanence of these breeding sites during the rainy season.

In Serrambi, the *Biomphalaria* are distributed in different locations, but climatic variations can helps the accidental confrontation between the two species, thus initiating the phenomenon of competitive exclusion. In the area predominate the natural breeding sites such as puddles. These, although located in the peridomicile, can only contribute to the transmission of the disease if climatic conditions lead to the occurrence of floods in the locality, transporting these breeding sites to the streets and forcing the population to expose themselves when leaving their houses. Barbosa et al.^7^ discuss this seasonal model of exposure to disease in coastal areas where residential sewage is dumped into open ditches in the peridomicile, providing the intermediate host snails infectivity which reproducing there. In the rainy season, these ditches overflow, taking the infected snails into the streets and backyards, where people become infected by contact with the contaminated water.

The relationship between elevation (DEM) and rainy season was observed in this study, showing that the lower slope area suffer the influence of precipitation, causing the accumulation of water, which is an aggravating factor for the disease epidemiology and transmission. Barbosa et al.^11^, using a distance map and a water expansion map, found an association between patients with schistosomiasis and the proximity to breeding sites or foci of *Biomphalaria* in Porto de Galinhas, municipality of Ipojuca, Pernambuco. In Itamaracá, the analysis performed through the distance maps showed that the main centers of active transmission of the disease are located in the streets around schools and local helthcare center, places of compulsory and systematic access by the population. During the rainy season, residents may become infected as they walk through these flooded streets filled with infected *S. mansoni intermediate host* snails. In Serrambi, the foci are located in the peridomicile and the fecal contamination of these environments will complete the disease cycle and may include the area as another coastal locality of autochthonous transmission of schistosomiasis.

It is important to note that the most efficient method used for diagnosis the snails in this study was the Nested PCR; however, this technique is not able to determine the rate of positivity, considering that methodology is applied to a pool of snails, constituting a limitation of our study.

In this study, the techniques of collection and analysis using geoprocessing proved to be important tools for location and scaling the risk areas for schistosomiasis and could support planning and contribute to the timely application of control measures by the health services. The health services can use this instrument for surveillance and monitoring the disease in localities where residents and tourists are exposed to unhealthy environments.
